# Low Bone Turnover Markers in Young and Middle-Aged Male Patients with Type 2 Diabetes Mellitus

**DOI:** 10.1155/2020/6191468

**Published:** 2020-08-10

**Authors:** X. X. Liu, L. Jiang, Q. Liu, J. Zhang, W. Niu, J. Liu, Q. Zhang

**Affiliations:** NHC Key Laboratory of Hormones and Development, Tianjin Key Laboratory of Metabolic Diseases, Chu Hsien-I Memorial Hospital & Tianjin Institute of Endocrinology, Tianjin Medical University, Tianjin 300134, China

## Abstract

Accumulating evidence has supported an increased risk of osteoporotic fracture in postmenopausal women and elderly men diagnosed with diabetes mellitus. However, it is not uncommon for young and middle-aged male patients diagnosed with type 2 diabetes mellitus (T2DM) to suffer from osteopenia or osteoporosis. Few studies focused on this population group are available. The aim of this study is to evaluate bone metabolic status and investigate the influence of T2DM on bone metabolism in 30-50-year-old men. Anthropometric assessment and blood samples were obtained from 160 patients with T2DM and 69 nondiabetic volunteers. Serum parathyroid hormone (PTH) and bone turnover markers (BTMs), including serum procollagen type I N-terminal peptide (PINP), osteocalcin (OC), and *β*-cross-linked C-telopeptide of type I collagen (*β*-CTX), were analysed. No significant differences were observed based on age, body mass index, systolic blood pressure, serum calcium, phosphorus, creatinine, total protein, and albumin levels when comparing T2DM and control groups. Fasting blood glucose, HbA1c, triglyceride (TG), total cholesterol, and low-density lipoprotein cholesterol were significantly increased, while high-density lipoprotein cholesterol was significantly decreased in the T2DM group. Compared with controls, diabetic patients showed lower serum PINP, OC, and PTH levels, whereas serum *β*-CTX levels were similar between the two groups. Moreover, HbA1c levels were positively correlated with PINP and inversely associated with PTH levels. TG levels were negatively correlated with OC or *β*-CTX levels. Furthermore, multiple linear regression revealed a positive correlation between HbA1c and PINP levels. These results also revealed a negative association between HbA1c and PTH, and between TG and OC levels, even after adjusting for expected confounder factors. Collectively, these findings indicated that young and middle-aged male patients with T2DM showed a lower turnover state resulting from bone formation inhibition. Glucose and lipid metabolic disorders may affect bone formation through different pathways.

## 1. Introduction

Type 2 diabetes mellitus (T2DM) is a common, chronic metabolic disease caused by insufficient insulin secretion and/or activity, leading to chronic hyperglycaemia. Its high prevalence has resulted in a heavy burden on social, financial, and health care systems [1]. There is a large amount of evidence revealing an increased risk of fracture in diabetic patients, particularly hip fracture [2, 3]. Recent meta-analyses indicated that hip fracture risk increases 1.3-1.7 times in patients with T2DM [4, 5]. In addition, studies had demonstrated that severe vertebral fracture in patients with T2DM was associated with increased all-cause mortality [6, 7]. Osteoporotic fracture has been increasingly recognized as another complication of T2DM. High morbidity and mortality make the two diseases be more serious global health problem. The association between osteoporosis and T2DM should be paid close attention.

Osteoporosis is a skeletal chronic metabolic disease characterized by low bone mass and destroyed bone microarchitecture, resulting in the high risk of fragility fracture [8]. Therefore, bone metabolism should be further studied in patients with T2DM. Bone metabolism is a dynamic cyclical process where osteoblasts are involved in bone formation, and osteoclasts are involved in bone resorption [9]. Metabolites known as bone turnovers markers (BTMs) are generated from bone tissue and cells during the dynamic process and reflect bone metabolism during a relatively short period of time [9] and are thus better at predicting more recent changes. Specifically, procollagen type I N-terminal peptide (PINP) is the degradation product during the formation of type I collagen secreted by osteoblasts; serum osteocalcin (OC) is released by osteoblasts during bone formation; *β*-cross-linked C-telopeptide of type I collagen (*β*-CTX) is a breakdown product during the degradation of mature type I collagen secreted by osteoclasts [9]. Consequently, PINP and OC are key markers of bone formation and *β*-CTX is a key marker for bone resorption. The International Osteoporosis Foundation (IOF) recommends PINP and *β*-CTX as the reference markers for bone formation and bone resorption, respectively, due to their high sensitivity and specificity [10]. Recently, these BTMs have been used to assess bone metabolism, evaluate the clinical efficacy of osteoporosis therapies, and predict fracture risk [11]. Additionally, BTMs are shown to be associated with energy metabolism [12], which is closely related to glucose metabolism. Studying the effect of glucose metabolism disorders on BTMs is important to evaluate bone metabolic status in T2DM.

Most research has focused on studying postmenopausal women and elderly men since these two groups of individuals are at a high risk for fractures, especially those diagnosed with T2DM. Bone formation and bone mass are highest in the third decade and then decrease with age [13, 14]. However, osteopenia or osteoporosis in young and middle-aged male patients with T2DM is not uncommon in clinical practice. Yet, only a few studies focused on these population groups are available. It is important to study how bone metabolism disorders affect younger patients with T2DM. Therefore, young and middle-aged male patients with T2DM were recruited as the subjects in the study presented here. We aim to assess bone metabolism by determining serum PINP, OC, *β*-CTX, and parathyroid hormone (PTH) levels and investigate the association among these markers and glucose metabolism. The goal is to explore the influence of T2DM on bone metabolism, which may allow for an accurate assessment of fracture risk and an earlier management of bone metabolism disorders.

## 2. Materials and Methods

### 2.1. Participants

The study presented here is a cross-sectional study conducted in men aged 30-50 years old. Patients with T2DM, who were admitted to the Tianjin Metabolic Diseases Hospital from December 2017 to February 2018, were included in the T2DM group. Nondiabetic, male volunteers from the physical examination centre were recruited and included in the control group during the same period.

The diagnosis of T2DM was based on the guidelines provided by the World Health Organization [15] including: fasting blood glucose (FBG) level ≥ 7.0 mmol/l (126 mg/dl) or 2 h blood glucose ≥ 11.1 mmol/l (199 mg/dl) during an oral glucose tolerance test (OGTT). Diabetic patients were treated with oral antidiabetic agents or in combination with insulin. Exclusion criteria included the presence of kidney disease (eGFR < 60 mL/min/1.73 m^2^), hepatic disease (ALT or AST ≥ 2.5 times than the upper reference), cancer, rheumatic diseases (rheumatic arthritis and rheumatoid arthritis), other bone metabolic diseases (osteitis and osteomalacia), hypercalcemia, or other endocrine diseases (Cushing's syndrome, primary hyperparathyroidism, and thyroid dysfunction). Participants taking medications that may influence bone metabolism were also excluded. These medications included glucocorticoids, calcium, vitamin D, antiosteoporosis drugs, steroids, and thyroid hormones.

This study was conducted following the Declaration of Helsinki (1964) and was approved by the Ethics Committee of the Tianjin Medical University Chu Hsien-I Memorial Hospital. Each participant signed a written informed consent form.

### 2.2. Clinical Measurements

Anthropometric and biochemical assessments were performed in all participants. Diabetic duration, height, weight, body mass index (BMI), and blood pressure data were collected. BMI was calculated by the formula as weight (in kg) divided by height squared (in m^2^). All overnight fasting blood samples were obtained in the morning. Serum samples were separated by centrifugation and stored at -80°C. Blood calcium, phosphorus, total protein, albumin, alanine aminotransferase, aspartate aminotransferase, alkaline phosphatase (ALP), serum creatinine, uric acid, urea nitrogen, haemoglobin A1c (HbA1c), FBG, insulin, C-peptide, total cholesterol (TC), triglyceride (TG), high-density lipoprotein cholesterol (HDL-c), and low-density lipoprotein cholesterol (LDL-c) were measured using standard methods. Serum parathyroid hormone (PTH) and BTM levels, including PINP, OC, and *β*-CTX, were measured using an IDS-iSYS automated analyser (Roche, Germany). The intra-assay and interassay coefficients of variation (CVs) of BTMs were below 5% and 7%, respectively.

### 2.3. Statistical Analyses

The statistical analyses were performed with SPSS 20.0 (SPSS Inc., Chicago, IL, USA). Normality testing was conducted in all continuous variables. Variables with normal distributions were described as mean ± standard deviation, and the differences were determined using Student's *t*-test between the two groups. Those with skewed distributions were expressed as median (interquartile range), and differences between groups were assessed using the Mann–Whitney *U* test. The Pearson or Spearman correlation analysis was used to determine the correlation between blood glucose or lipid and bone metabolism markers. Multiple linear regression analyses were conducted to evaluate the association between HbA1c, TG, and BTMs. *P* value < 0.05 was considered statistically significant.

## 3. Results

A total of 160 diabetic patients were included in the T2DM group. The mean age of these patients was 41.0 ± 5.9 years and the mean diabetic duration was 4.0 years (ranging from 1.0 to 9.0 years). The mean FBG was 8.64 ± 2.40 mmol/l, and the mean HbA1c value was 8.69 ± 1.84%. A total of 69 nondiabetic volunteers were recruited in the control group that had a mean age of 41.8 ± 5.1 years and a mean FBG of 5.09 ± 0.27 mmol/l.

Baseline clinical characteristics of the two groups are shown in Table 1. No significant differences were observed between the control or T2DM groups for age (*P* = 0.285), height (*P* = 0.220), weight (*P* = 0.097), BMI (*P* = 0.294), or systolic blood pressure (*P* = 0.347). There were also no significant differences between the two groups for serum calcium (*P* = 0.193), phosphorus (*P* = 0.118), creatinine (*P* = 0.639), total protein (*P* = 0.897), albumin (*P* = 0.825), or ALP (*P* = 0.410). As expected, patients in the T2DM group showed significantly higher FBG levels (*P* < 0.001) compared with the control group. In addition, significantly higher TG (*P* < 0.001), TC (*P* < 0.001), and LDL-c (*P* < 0.001) levels and significantly lower HDL-c (*P* = 0.020) levels were observed in diabetic patients compared with controls.

Comparison of BTMs and PTH between diabetic patients and controls is shown in Table 2. There were significant decreases in serum PINP (*P* = 0.008), OC (*P* < 0.001), and PTH (*P* = 0.001) levels in patients with T2DM compared with controls. In contrast, serum *β*-CTX levels were similar between the two groups (*P* = 0.826).

**Figure 1:**
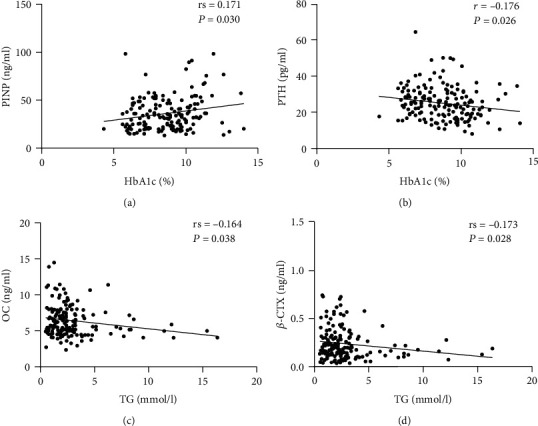
Correlation between serum glucose or lipid levels and BTMs or PTH. (a) Correlation between PINP and HbA1c. (b) Correlation between PTH and HbA1c. (c) Correlation between OC and TG. (d) Correlation between *β*-CTX and TG. HbA1c: haemoglobin A1c; TG: triglyceride; PINP: procollagen type I N-terminal peptide; OC: osteocalcin; *β*-CTX: *β*-cross-linked C-telopeptide of type I collagen; PTH: parathyroid hormone. *r*: Pearson's correlation coefficient; rs: Spearman's correlation coefficient. *P* value < 0.05 was considered significant.

Moreover, univariate correlation analyses were performed to investigate the association between blood glucose or lipid and bone metabolism markers. The results revealed that HbA1c was positively correlated with PINP (rs = 0.171, *P* = 0.030) and inversely associated with PTH (*r* = −0.176, *P* = 0.026). There was a significant negative correlation between OC or *β*-CTX and TG (rs = −0.164, *P* = 0.038; rs = −0.173, *P* = 0.028) levels (Figure 1). There was no significant association observed between PINP and TG, or between OC and HbA1c levels. Age was negatively correlated with PINP (rs = −0.269, *P* = 0.001), OC (rs = −0.168, *P* = 0.033), and PTH (*r* = 0.164, *P* = 0.038), but not with *β*-CTX levels. The BTMs and PTH levels did not correlate with BMI, blood pressure, calcium, or phosphorous levels.

Furthermore, multiple linear regression analyses were performed to examine the cross-sectional association between blood glucose or lipid and BTMs after adjusting for expected confounder factors. Serum PINP, OC, or PTH levels were used as dependent variables, while HbA1c or TG levels were used as independent variables. These findings indicated that HbA1c was positively correlated with PINP (*β* = 0.229, *P* = 0.004) and inversely associated with PTH (*β* = −0.213, *P* = 0.011) after adjusting for age, BMI, systolic blood pressure, TG, HDL-c, LDL-c, calcium, and phosphorus. Our results also showed a significant negative correlation between TG and OC (*β* = −0.177, *P* = 0.034) after adjusting for age, BMI, systolic blood pressure, HbA1c, HDL-c, LDL-c, calcium, and phosphorus (Table 3). All independent variables used in multiple linear analyses are shown in Table S1.

## 4. Discussion

Most previous studies investigating postmenopausal women and elderly men have shown that the markers for bone formation and/or resorption were reduced in patients with T2DM, compared with nondiabetic individuals [16], indicating a lower bone turnover state. It is unclear whether young and middle-aged diabetic patients shared similar results. In this study, we focused on young and middle-aged male patients with T2DM. Results demonstrated that diabetic patients had significantly lower serum PINP and OC levels compared with the control individuals. In contrast, serum *β*-CTX levels were not significantly different between the two groups. Results indicated that inhibition of bone formation phase, rather than resorption, led to a lower bone turnover state in young and middle-aged male patients with T2DM. Moreover, this study demonstrated that HbA1c was an independent factor for PINP, suggesting the influence of glycaemic control on PINP in young and middle-aged male patients with T2DM. Early glycaemic control may reduce the risk of fracture by delaying bone formation reduction.

Reduced serum OC levels were previously reported in male patients with T2DM [17–19]. Bezerra dos Santos Magalhaes et al. further demonstrated a weak negative correlation between FBG and OC levels [19]. Whereas serum PINP was not available in these studies. A recent study revealed a decrease in serum PINP levels in patients with impaired fasting glucose and diabetes [20], which was in line with our research. Further analyses revealed that serum PINP levels were significantly reduced in younger diabetic patients (<65 years old) compared with the older patients (≥65 years old), but serum *β*-CTX was also significantly decreased [20]. The controversial conclusions may be related to differences in age, race, diabetic duration, and glycaemic control. A study by Kulkarni et al. [18] shared a similar relationship between HbA1c and PINP levels. Additionally, a large-scale cross-sectional study performed in Germany indirectly supported this conjecture. The authors revealed that chances for metabolic syndrome or T2DM significantly decreased with the higher serum PINP and *β*-CTX levels in men aged 25-55 years old [21]. However, two large-scale studies performed in China, one involving men and women aged 30-60 years old [22] and the other including men aged 20-69 years old [23], indicated that serum OC was negatively correlated with chances for T2DM, even after adjusting age, BMI, waist circumference, blood pressure, FBG, and TG. As described by these studies, the close relationship between glucose and BTMs has been investigated but needs further understanding.

In addition, compared with controls, diabetic patients showed higher TG, TC, and LDL-c and lower HDL-c levels, which may represent a high probability of lipid metabolism disorders in patients with T2DM. Further analyses investigating the correlation between lipid and BTMs revealed a significant, negative correlation between serum TG and OC levels. High TG levels may reduce serum OC levels and inhibit bone formation in young and middle-aged male patients with T2DM. These observations were similar to what was found in a recent male population-based study where serum TG levels were also inversely correlated with OC levels [23]. Some research investigating male diabetic patients showed no relationship between serum OC levels and blood lipid metabolism [24, 25]. These findings are contradictory to one another. Differences in age, race, and metabolic status may account for these controversial results.

The impact of blood glucose and lipid metabolism disorders on BTMs needs further studies to elucidate mechanisms. It is known that hyperglycaemia can lead to osmotic diuresis, which causes renal calcium leakage and a negative calcium balance. Improved blood glucose control contributes to the reduction of urinary calcium levels [26]. The calcium-sensing defect and secondary chronic hypomagnesaemia induced by osmotic diuresis may be responsible for impaired PTH secretion [27]. The pathological regulation of PTH on BTMs in patients with T2DM is not clear. In this study, serum PTH levels were decreased and were negatively associated with HbA1c levels in T2DM, implying that diabetic patients, especially those with poor glycaemic control, had lower PTH levels. These observations were in line with previous studies [28, 29]. Relative hypoparathyroidism may disrupt bone metabolism in patients with T2DM. Previous studies demonstrated that low PTH levels directly inhibited osteoblast activity and contribute to bone demineralization. In the nondiabetic population, PTH inhibited transcriptional suppression of sclerostin produced by osteocytes. As a Wnt antagonist, sclerostin inhibited Wnt/*β*-catenin signalling and osteoblast activity. However, the regulation of PTH on sclerostin may be impaired in diabetes [30]. As mentioned above, the negative relationship between blood glucose and bone metabolism is probably explained.

Otherwise, chronic inflammatory conditions and turbulence of adipokines increased the risk of osteoporosis in patients with T2DM [31]. Advanced glycation end-products (AGEs) were accumulated in diabetes and played a key role in chronic inflammatory complications [32]. Previous studies have shown that BTMs were suppressed by hyperinsulinemia and the accumulation of AGEs [33]. AGEs promoted the production of both inflammatory cytokines and reactive oxygen species (ROS) in the body by activating ligands, further triggering chronic inflammation and bone resorption [34]. *In vitro* studies reported that AGE-2 and AGE-3 inhibited the maturation of human marrow mesenchymal stem cells (MSCs) and their differentiation into cartilage and bone tissues, resulting in decreased osteoblasts [35]. Moreover, the formation and accumulation of AGEs inhibited synthesis of osteocalcin and osteoblastic ossein matrix [36], increased nonenzymatic cross-linked folding of the collagen fibres [37], and disturbed osteoblast development. A recent study indicated that hyperglycaemia directly inhibited the differentiation of osteoblasts and then decreased bone formation, enhanced osteoclast activity, and increased bone absorption, eventually leading to a reduction of bone mass [38, 39]. Glucose and insulin signalling involved receptor activation of the nuclear factor *κ*B ligand/osteoprotegerin (RANKL/OPG) pathway [40, 41]. Analyses revealed that lower serum RANKL levels were associated with higher TG levels [42]. This inverse relationship may explain the results generated in this study. Furthermore, adiponectin, a recently uncovered adipocytokine, is produced exclusivity in adipose tissue. Research shows that adiponectin stimulated osteoblast proliferation, differentiation, and mineralization [43]. However, serum adiponectin concentrations decreased in patients with T2DM [44]. The turbulence of adipocytokines may lead to an imbalance of bone metabolism.

Antidiabetic agents may have different effects on bone metabolism. Agents that may have an effect include thiazolidinediones (TZDs), sodium-glucose-linked transporter-2 (SGLT-2) inhibitors, insulin, and glucagon-like peptide-1 receptor agonists (GLP-1 RA). A previous work shows that rosiglitazone, a type of TZDs, promoted osteoblast/osteocyte apoptosis and led to a negative balance in bone metabolism [45]. Analyses demonstrated a gender difference when it came to the effects of TZDs on fracture in patients with T2DM and confirmed that TZDs only increased fracture risk in female patients and not male patients [46]. SGLT-2 inhibitors improved blood glucose levels by promoting urinary glucose excretion, which may affect urinary calcium excretion and bone metabolism. Canagliflozin treatment was associated with a higher fracture rate in patients with T2DM [47]. A meta-analysis indicated no relationship between three SGLT-2 inhibitors (canagliflozin, dapagliflozin, and empagliflozin) and fracture risk. Clinical studies on adverse skeletal events of SGLT-2 inhibitors are still lacking. Few studies have assessed the association between insulin injection and BTMs. Several studies reported an increased fracture risk in insulin-treated patients with T2DM [48]. A high incidence of hypoglycaemic events and falling [49] may be the main reasons in older adults. Long-term disease and the presence of multiple diabetic complications may also disrupt bone metabolism. No significant differences were observed between diabetic patients under treatment with (*n* = 35) or without (*n* = 125) TZDs, with (*n* = 10) or without (*n* = 150) SGLT-2 inhibitors, and with (*n* = 85) or without (*n* = 75) insulin in this study. Liraglutide and exenatide, two GLP-1 RAs, may improve skeletal blood supply, increase bone mineral density (BMD), and reduce the risk of osteoporosis and fracture in animal and human studies [27, 50]. However, the bone protective effects behind this require clinical studies. There were no significant differences observed between diabetic patients under treatment with (*n* = 12) or without (*n* = 148) GLP-1 RAs in our study. In addition, there were also no significant differences between patients under treatment with (*n* = 54) or without (*n* = 106) dipeptidyl peptidase-4 (DPP-4) inhibitors, with (*n* = 42) or without (*n* = 118) insulin secretagogues, with (*n* = 108) or without (*n* = 52) metformin, and with (*n* = 94) or without (*n* = 66) alpha-glucosidase inhibitors (AGI) in the present study (Table S2). As a multiple metabolic disease, the treatment of T2DM is complex and requires additional clinical studies to evaluate the influence of these therapies on bone metabolism.

One advantage of this study is that it focused on male diabetic patients aged 30-50 years old, where BTMs varied with small changes and there was a restriction on gender and age being an influence on the results. With this, it was easier to investigate the relationship between blood glucose, lipids, and bone metabolism. However, this study still faces some limitations. First, the cross-sectional design prevents one from drawing a causal relationship and failed to explore changes in BTMs after improving blood glucose and lipid metabolism disorders. Further prospective research may offer additional information about this. Second, the sample size number between the two groups was unequal and the number of controls used was inadequate. Besides, this study was a single-centre study that only analysed patients with relatively severe diabetes. Therefore, the results presented here may not be generalizable to all young and middle-aged male populations diagnosed with T2DM. Large-scale and multicentre studies remained to verify these issues. Third, the influence of antidiabetic agents on bone metabolism remains contradictory. Consequently, potential confounder factors may exist. Fourth, serum levels of bone-specific alkaline phosphatase (BAP), vitamin D, or steroids, all of which influence bone metabolism, were not determined in this study. Serum ALP is mainly derived from liver isoform (LAP), and its specificity for bone metabolism is lacking [51]. BAP is a more bone-specific marker of bone formation, while the current immunoassays available for BAP still show up to 20% cross-reactivity toward LAP [52]. As recommended by the IOF [10], serum PINP was preferred for bone formation because of high specificity in our study. Vitamin D promotes the absorption of calcium and may affect bone metabolism. However, relatively limited data about the effect of vitamin D on BTMs are available. A prospective partial intervention study in postmenopausal women with T2DM shows that 25(OH)D was positively correlated with PINP, especially in patients taking alfacalcidol [53]. The MINOS study, a prospective study of 881 men aged 19-85 years, revealed that serum 25(OH)D was not associated with BTMs in men under 55 years of age (*n* = 286) [54]. The relationship between vitamin D and BTMs still needs further research. Fifth, we did not take BMD into consideration. BMD altogether with BTMs may be helpful to evaluate bone metabolism. BMD reflects mineral density of bone and is the cumulative result of long-term bone metabolic activities. This study mainly focused on the impact of T2DM on BTMs and evaluated the recent changes of bone metabolism. Further studies should be conducted to investigate the long-term effect of T2DM on BMD.

## 5. Conclusions

This study demonstrated that young and middle-aged male patients with T2DM showed a lower turnover state resulting from bone formation inhibition. HbA1c levels were positively correlated with PINP levels and inversely associated with PTH levels. These findings also revealed a negative correlation between TG and OC levels, even after adjusting for expected confounder factors. Glucose and lipid metabolism disorders may affect bone formation through different pathways. The study presented here provides evidence of T2DM influencing bone metabolism in young and middle-aged men. The improvement of blood glucose and lipids may be beneficial to bone metabolism and reduce fracture risk in patients with T2DM.

## Figures and Tables

**Table 1:** Comparison of characteristics between diabetic patients and controls.

Variables	Patients with T2DM (*n* = 160)	Nondiabetic controls (*n* = 69)	*P*
Age (y)	41.0 ± 5.9	41.8 ± 5.1	0.285
Diabetic duration (y)	4.0 (1.0, 9.0)	—	—
Height (cm)	173.7 ± 6.7	172.8 ± 4.5	0.220
Weight (kg)	84.4 ± 15.0	81.4 ± 11.1	0.097
BMI (kg/m^2^)	27.89 ± 4.28	27.27 ± 3.68	0.294
SBP (mmHg)	129.6 ± 13.0	127.8 ± 13.0	0.347
DBP (mmHg)	80.8 ± 9.6	79.8 ± 9.0	0.470
FBG (mmol/l)	8.64 ± 2.40	5.09 ± 0.27	<0.001
HbA1c (%)	8.69 ± 1.84	—	—
INS (mIU/l)	9.28 (5.32, 13.73)	—	—
C-P (ng/ml)	1.59 (1.15, 2.16)	—	—
TG (mmol/l)	2.32 (1.42, 3.23)	1.22 (0.85, 1.86)	<0.001
TC (mmol/l)	5.08 ± 1.12	4.00 ± 0.94	<0.001
HDL-c (mmol/l)	1.06 ± 0.24	1.16 ± 0.33	0.020
LDL-c (mmol/l)	3.20 ± 0.96	2.43 ± 0.78	<0.001
Ca (mmol/l)	2.31 ± 0.13	2.33 ± 0.12	0.193
P (mmol/l)	1.31 ± 0.18	1.26 ± 0.18	0.118
TP (g/l)	68.70 ± 4.22	68.78 ± 5.10	0.897
ALB (g/l)	43.98 ± 3.17	44.08 ± 2.89	0.825
ALT (IU/l)	21.35 ± 9.78	20.97 ± 9.04	0.782
AST (IU/l)	20.42 ± 10.62	19.59 ± 7.15	0.554
ALP (IU/l)	70.9 ± 18.37	68.85 ± 14.92	0.410
Cr (*μ*mol/l)	71.04 ± 12.90	71.71 ± 8.25	0.639
SUA (*μ*mol/l)	344.45 ± 90.21	339.44 ± 60.07	0.932
BUN (mmol/l)	5.26 ± 1.17	5.19 ± 0.87	0.641

y: years; T2DM: type 2 diabetes mellitus; BMI: body mass index; SBP: systolic blood pressure; DBP: diastolic blood pressure; FBG: fasting blood glucose; HbA1c: haemoglobin A1c; INS: fasting insulin; C-P: fasting C-peptide; TG: triglyceride; TC: total cholesterol; HDL-c: high-density lipoprotein cholesterol; LDL-c: low-density lipoprotein cholesterol; Ca: calcium; P: phosphorus; TP: total protein; ALB: albumin; ALT: alanine aminotransferase; AST: aspartate aminotransferase; ALP: alkaline phosphatase; Cr: serum creatinine; SUA: serum uric acid; BUN: blood urea nitrogen. *P* value < 0.05 was considered significant.

**Table 2:** Comparison of BTMs and PTH between diabetic patients and controls.

Variables	Patients with T2DM	Nondiabetic controls	*P*
PINP (ng/ml)	33.38 (22.53, 46.02)	41.33 (30.40, 48.51)	0.008
OC (ng/ml)	5.74 (4.92, 7.55)	7.14 (5.64, 9.18)	<0.001
*β*-CTX (ng/ml)	0.19 (0.12, 0.30)	0.20 (0.14, 0.27)	0.826
PTH (pg/ml)	25.21 ± 9.13	29.86 ± 11.13	0.001

T2DM: type 2 diabetes mellitus; PINP: procollagen type I N-terminal peptide; OC: osteocalcin; *β*-CTX: *β*-cross-linked C-telopeptide of type I collagen; PTH: parathyroid hormone. *P* value < 0.05 was considered significant.

**Table 3:** Multiple linear regression analyses between serum glucose or lipid and bone metabolism markers.

Dependent variable	Independent variables	Unstandardized coefficients (*β*)	Standardized coefficients (*β*)	*P*
PINP	HbA1c	2.140	0.229	0.004
OC	TG	-0.144	-0.177	0.034
PTH	HbA1c	-1.054	-0.213	0.011

HbA1c: haemoglobin A1c; TG: triglyceride; PINP: procollagen type I N-terminal peptide; OC: osteocalcin; PTH: parathyroid hormone. *P* value < 0.05 was considered significant.

## Data Availability

The data used to support the findings of this study are available from the corresponding author upon request.

## Supplementary Materials

Supplementary Materials Table S1: multiple linear regression between serum glucose or lipid levels and BTMs or PTH. Table S2: the information about the medications of the patients with T2DM.
