# Supplementary material for: Low Bone Turnover Markers in Young and Middle-Aged Male Patients with Type 2 Diabetes Mellitus
Source: J Diabetes Res. 2020 Aug 10;2020:6191468. doi: 10.1155/2020/6191468 (PMC7436354; doi:10.1155/2020/6191468)
Supplement: Supplementary Materials — Table S1: multiple linear regression between serum glucose or lipid levels and BTMs or PTH. Table S2: the information about the medications of the patients with T2DM. [file 6191468.f1.docx]

**Supplementary Material**

**Supplementary Table 1:** Multiple linear regression between serum glucose or lipid levels and BTMs or PTH.

| **Dependent variable** | **Independent variables** | **Unstandardized coefficients (β)** | **Standardized coefficients (β)** | **P** |
| --- | --- | --- | --- | --- |
| **PINP** | age | -0.308 | -3.985 | ＜0.001 |
|  | BMI | 0.026 | 0.322 | 0.748 |
|  | SBP | -0.055 | -0.696 | 0.487 |
|  | HbA1c | 0.229 | 2.918 | 0.004 |
|  | TG | -0.071 | -0.893 | 0.373 |
|  | HDL-c | 0.115 | 1.402 | 0.163 |
|  | LDL-c | 0.128 | 1.659 | 0.099 |
|  | calcium | -0.054 | -0.698 | 0.486 |
|  | phosphorus | 0.138 | 1.831 | 0.069 |
| **OC** | age | -0.210 | -2.619 | 0.010 |
|  | BMI | 0.039 | 0.467 | 0.641 |
|  | SBP | -0.085 | -1.037 | 0.301 |
|  | HbA1c | -0.015 | -0.190 | 0.850 |
|  | TG | -0.177 | -2.141 | 0.034 |
|  | HDL-c | 0.110 | 1.286 | 0.200 |
|  | LDL-c | 0.139 | 1.740 | 0.084 |
|  | calcium | -0.096 | -1.207 | 0.230 |
|  | phosphorus | 0.099 | 1.263 | 0.209 |
| **PTH** | age | 0.196 | 2.414 | 0.017 |
|  | BMI | 0.044 | 0.529 | 0.597 |
|  | SBP | 0.139 | 1.685 | 0.094 |
|  | HbA1c | -0.213 | -2.587 | 0.011 |
|  | TG | -0.063 | -0.750 | 0.455 |
|  | HDL-c | -0.036 | -0.420 | 0.675 |
|  | LDL-c | -0.039 | -0.481 | 0.631 |
|  | calcium | -0.107 | -1.326 | 0.187 |
|  | phosphorus | -0.116 | -1.466 | 0.145 |

PINP: procollagen type I N-terminal peptide; OC: osteocalcin; PTH: parathyroid hormone; BMI: body mass index; SBP: systolic blood pressure; HbA1c: haemoglobin A1c; TG: triglyceride; HDL-c: high-density lipoprotein cholesterol; LDL-c: low-density lipoprotein cholesterol. P value < 0.05 was considered significant.

**Supplementary Table 2:** The information about the medications of the patients with T2DM.

| **anti-diabetic agents** | | **PINP** | | **OC** | | **β-CTX** | | **PTH** | |
| --- | --- | --- | --- | --- | --- | --- | --- | --- | --- |
|  |  | **ng/ml** | **P** | **ng/ml** | **P** | **ng/ml** | **P** | **pg/ml** | **P** |
| **TZDs** | |  |  |  |  |  |  |  |  |
|  | **yes (n = 35)** | 28.63 (20.15, 46.02) | 0.426 | 5.71 (4.79, 8.02) | 0.700 | 0.20 (0.13, 0.34) | 0.346 | 25.85 ± 9.30 | 0.627 |
|  | **no (n = 125)** | 33.90 (23.82, 46.33) |  | 5.74 (4.94, 7.32) |  | 0.19 (0.12, 0.30) |  | 25.00 ± 9.11 |  |
| **SGLT-2i** | |  |  |  |  |  |  |  |  |
|  | **yes (n = 10)** | 31.92 (19.16, 47.68) | 0.677 | 5.87 (4.56, 8.88) | 0.789 | 0.21 (0.10, 0.54) | 0.698 | 26.77 ± 11.04 | 0.572 |
|  | **no (n = 150)** | 33.90 (22.60, 46.18) |  | 5.74 (4.93, 7.43) |  | 0.19 (0.12, 0.30) |  | 25.08 ± 9.02 |  |
| **GLP-1 RA** | |  |  |  |  |  |  |  |  |
|  | **yes (n = 12)** | 33.45 (21.86, 52.77) | 0.821 | 5.60 (4.89, 6.64) | 0.693 | 0.20 (0.09, 0.35) | 0.902 | 23.33 ± 7.32 | 0.466 |
|  | **no (n = 148)** | 33.38 (22.53, 45.84) |  | 5.74 (4.92, 7.56) |  | 0.19 (0.12, 0.30) |  | 25.33 ± 9.26 |  |
| **DPP-4i** | |  |  |  |  |  |  |  |  |
|  | **yes (n = 54)** | 32.53 (22.49, 45.49) | 0.705 | 6.10 (4.99, 7.52) | 0.573 | 0.19 (0.12, 0.28) | 0.649 | 26.01 ± 8.36 | 0.416 |
|  | **no (n = 106)** | 33.90 (22.38, 46.78) |  | 5.63 (4.89, 7.63) |  | 0.19 (0.12, 0.32) |  | 24.76 ± 9.51 |  |
| **metformin** | |  |  |  |  |  |  |  |  |
|  | **yes (n = 108)** | 33.38 (23.85, 45.09) | 0.938 | 5.68 (4.62, 7.46) | 0.265 | 0.18 (0.12, 0.30) | 0.229 | 25.68 ± 8.86 | 0.227 |
|  | **no (n = 52)** | 33.90 (20.17, 49.91) |  | 6.21 (4.97, 7.66) |  | 0.23 (0.14, 0.30) |  | 23.81 ± 9.43 |  |
| **insulin secretagogues** | |  |  |  |  |  |  |  |  |
|  | **yes (n = 42)** | 31.90 (20.07, 42.60) | 0.312 | 5.70 (4.89, 7.07) | 0.548 | 0.17 (0.11, 0.30) | 0.443 | 23.52 ± 8.51 | 0.170 |
|  | **no (n = 118)** | 34.04 (23.69, 46.65) |  | 5.81 (4.94, 7.77) |  | 0.20 (0.13, 0.31) |  | 25.77 ± 9.30 |  |
| **AGI** | |  |  |  |  |  |  |  |  |
|  | **yes (n = 94)** | 32.77 (22.60, 44.47) | 0.607 | 5.57 (4.77, 7.22) | 0.250 | 0.20 (0.13, 0.30) | 0.890 | 24.80 ± 9.42 | 0.528 |
|  | **no (n = 66)** | 34.69 (22.05, 48.64) |  | 6.27 (4.97, 7.90) |  | 0.19 (0.12, 0.30) |  | 25.73 ± 8.74 |  |
| **Insulin injection** | |  |  |  |  |  |  |  |  |
|  | **yes (n = 85)** | 32.46 (23.55, 46.65) | 0.604 | 5.45 (4.75, 7.36) | 0.417 | 0.20 (0.12-0.30) | 0.863 | 24.99 ± 10.09 | 0.782 |
|  | **no (n = 75)** | 34.69 (21.31, 45.31) |  | 6.23 (4.97, 7.63) |  | 0.19 (0.13-0.30) |  | 25.40 ± 7.96 |  |

T2DM: type 2 diabetes mellitus; PINP: procollagen type I N-terminal peptide; OC: osteocalcin; β-CTX: β-cross-linked C-telopeptide of type I collagen; PTH: parathyroid hormone; TZDs: thiazolidinediones; SGLT-2i: sodium-glucose linked transporter-2 inhibitors; GLP-1 RA: glucagon-like peptide-1 receptor agonists; DPP-4i: dipeptidyl peptidase-4 inhibitors; AGI: alpha-glucosidase inhibitors. Insulin secretagogues included sulfonylureas and glinides. P value < 0.05 was considered significant.
